# Early Three-Dimensional Intrableb Structural Changes in Primary-Open Angle Glaucoma and Exfoliation Glaucoma After Ex-PRESS Surgery

**DOI:** 10.1167/tvst.11.2.32

**Published:** 2022-02-22

**Authors:** Ikki Sugimoto, Shinichi Usui, Tomoyuki Okazaki, Rumi Kawashima, Atsuya Miki, Ryo Kawasaki, Kenji Matsushita, Kohji Nishida

**Affiliations:** 1 Department of Ophthalmology, Osaka University Graduate School of Medicine, Osaka, Japan; 2 Osaka Rosai Hospital, Osaka, Japan; 3 Integrated Frontier Research for Medical Science Division, Institute for Open and Transdisciplinary Research Initiatives, Osaka University, Osaka, Japan

**Keywords:** exfoliation glaucoma, bleb, AS-OCT, filtration surgery, Ex-PRESS

## Abstract

**Purpose:**

To compare early-stage intrableb structural changes after Ex-PRESS surgery using anterior-segment optical coherence tomography between primary open-angle glaucoma (POAG) and exfoliation glaucoma (XFG).

**Methods:**

Twenty-five POAG eyes and 15 XFG eyes that underwent Ex-PRESS surgery were evaluated. Intrableb images were classified into four categories based on previously reported scattering intensity: high-, medium-, and low-scattering walls and fluid-filled spaces. Bleb measurements were evaluated in both groups throughout 6 postoperative months. The 3-year surgical success was defined by the criteria intraocular pressure (IOP) < 18 mmHg and IOP < 15 mmHg with or without medications.

**Results:**

The fluid-filled space volume at 3 and 6 months (*P* = 0.005 and *P* = 0.022, respectively) and the volume ratio of the low-scattering wall to the bleb wall (*P* = 0.028) at 6 months were significantly smaller in XFG than POAG postoperatively. The volume ratio of the high-scattering wall to the bleb wall was correlated positively (*P* = 0.007) with the IOP, and that of the low-scattering wall to the bleb wall was correlated negatively (*P* = 0.002) with the IOP in XFG. The 3-year surgical success rates for both criteria were significantly lower in XFG than POAG.

**Conclusions:**

Fluid-filled spaces were smaller in XFG than in POAG after Ex-PRESS surgery. The proportion of the high-scattering wall tended to increase and the low-scattering wall tended to decrease in XFG eyes with high IOP. Early-stage intrableb structural changes differed between POAG and XFG and may affect the prognosis.

**Translational Relevance:**

Our cutting-edge observation of intrableb fibrosis can be an important predictor of the surgical outcome.

## Introduction

Exfoliation glaucoma (XFG), caused by a disorder of the abnormal production and accumulation of extracellular elastin-related microfibrillar materials,^1^ is a severe vision-threatening glaucoma that occurs worldwide and differs from primary-open angle glaucoma (POAG) in clinical appearance, course, and prognosis.^2^^,^^3^ Because of high peak intraocular pressure (IOP)^4^ and wide IOP fluctuations, progression of visual field loss in XFG is more rapid than in POAG.^5^^,^^6^ Thus, XFG often requires surgical intervention when medical therapy fails.^7^ Filtration surgeries such as trabeculectomy^8^ and implantation of the Ex-PRESS Glaucoma Filtration Device (Alcon Laboratories, Fort Worth, TX)^9^^–^^11^ are the most effective surgeries with a wide range of IOP reduction. The surgical outcomes of filtration surgery for XFG are similar to those of POAG.^12^^–^^14^ In contrast, long-term IOP control of XFG has been reported to be poorer than in POAG.^15^^–^^18^ Several recent studies of bleb analysis using anterior-segment optical coherence tomography (AS-OCT) have been reported^18^^–^^25^; however, most compared successful and unsuccessful blebs in subjects with several glaucoma types using a cross-sectional study design. Therefore, the differences in the postoperative courses between POAG and XFG regarding bleb formation are not well known. The current study compared the early-stage intrableb structural changes in detail after Ex-PRESS surgery between POAG eyes and XFG eyes using AS-OCT.

## Methods

### Patients

This was a retrospective, consecutive case study. Patients with OAG were enrolled who met the inclusion criteria at the glaucoma clinic of Osaka University Hospital. From October 2012 to May 2017, three glaucoma surgeons (KM, SU, AM) performed Ex-PRESS insertion surgery in 111 eyes of 95 patients with glaucoma. We analyzed the three-dimensional (3D) findings of the blebs at 1, 3, and 6 months postoperatively using AS-OCT (CASIA SS-1000; Tomey, Nagoya, Japan) in 40 eyes (25 eyes of 25 patients in the POAG group and 15 eyes of 15 patients in the XFG group). Patients with eyes that met the following criteria were excluded: (1) eyes for which AS-OCT images had not been obtained at 1, 3, and 6 months postoperatively; (2) eyes that had undergone any internal eye surgery other than cataract surgery and trabeculotomy, additional glaucoma surgery, or needling during the study period or had bleb leaks after the second week postoperatively; and (3) eyes with poor-quality OCT images that contained artifacts or for which images were not obtained due to deep-set eyes. If both eyes met the selection criteria, only the eye treated first was evaluated. The same three glaucoma specialists evaluated the glaucomatous findings based on the best-corrected visual acuity, IOP, central corneal thickness, slit-lamp biomicroscopy, gonioscopy, dilated fundus examination, color fundus photographs of the optic disc, OCT images, and visual field tests using standard automated perimetry (SAP). Goldmann perimetry was performed for the patients for whom it was difficult to obtain SAP measurements because of advanced glaucoma or poor reliability resulting from fixation loss in the elderly. The institutional review board of Osaka University Hospital approved this observational study. The research adhered to the tenets of the Declaration of Helsinki.

### Ex-PRESS Implant Surgery and Management Postoperatively

The Ex-PRESS is a mini-glaucoma device that shunts aqueous humor from the anterior chamber to the subconjunctival space in a fashion similar to that of trabeculectomy. In all cases, a fornix-based conjunctival incision and single square scleral flap were created, and 0.04% mitomycin C (Kyowa Hakko Kirin Company, Tokyo, Japan) was applied for 3 minutes. A combined procedure that included lensectomy and intraocular lens (IOL) implantation was performed from another corneal wound if needed. Tenon tissue was covered in some cases with thin conjunctiva. In addition, laser suture lysis was performed appropriately postoperatively. Postoperative topical treatment with glaucoma eye drops was added based on the postoperative IOP, bleb formation, and degree of visual field loss.

### Evaluation of Intrableb Structure by 3D AS-OCT

**Figure 1.**
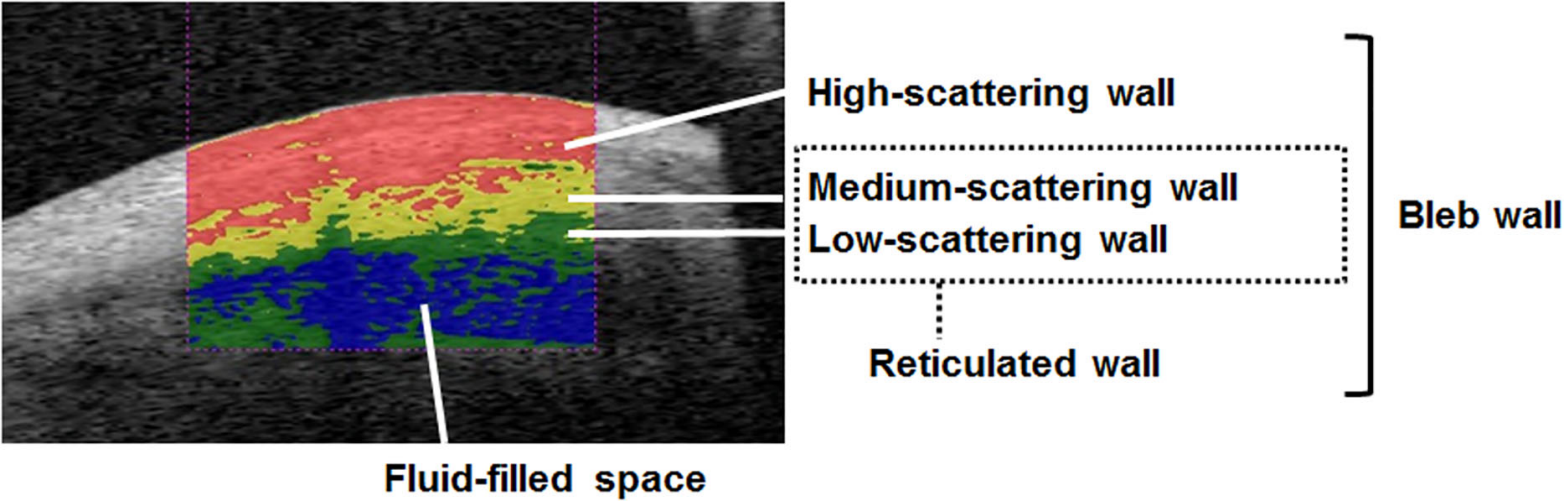
Intrableb structure by refractive categories. The intrableb structures are divided into four color-coded categories based on scattering intensity. *Red*, *yellow*, *green*, and *blue* indicate, respectively, the high-scattering wall, medium-scattering wall, low-scattering wall, and fluid-filled space. The intrableb structure is comprised of the bleb wall and fluid-filled space. The bleb wall is comprised of the high-scattering wall and reticulated wall. The reticulated wall is divided into the medium-scattering wall and low-scattering wall.

Filtration bleb images (12 × 12-mm square) were detected using the bleb mode of the 3D AS-OCT and evaluated using CASIA Bleb Assessment software, version 4.0L (Tomey), as reported previously.^23^^,^^25^ The bleb image was rotated so that the scleral flap was centered in the image, and the 3D image of the anterior bleb including the 3 × 3-mm square area behind the scleral flap was evaluated. The intrableb structural image was automatically divided into four color-coded categories based on the scattering intensity (scored from 0–255) previously reported. Red (scored from 150–255), yellow (scored from 100–149), green (scored from 50–99), and blue (scored from 0–49) indicate, respectively, the high-scattering wall, medium-scattering wall, low-scattering wall, and fluid-filled space.^23^^,^^25^ The intrableb structure consists of the bleb wall and fluid-filled space. The bleb wall was comprised of the high-scattering wall and reticulated wall, which was divided into the medium-scattering wall and low-scattering wall (Fig. 1). Each bleb measurement volume was compared between the POAG and XFG groups at 1, 3, and 6 months postoperatively. The relationships between these bleb measurements and the IOP were evaluated in both groups only in patients (about 87%) who did not receive additional glaucoma medication throughout the postoperative period.

### Medication Score

The number of glaucoma eye drops for treatment was defined as the medication score. A combination eye drop was counted as a score of 2.

### Surgical Success

Surgical success was defined as 5 mmHg < IOP < 18 mmHg with or without medications, and as >20% IOP reduction (criterion A), 5 mmHg < IOP < 15 mmHg with or without medications, and >20% IOP reduction (criterion B).^26^ The requirement for additional glaucoma surgeries, including needle bleb revision, was defined as complete failure.

### Other Examinations

A technician masked to the clinical diagnoses performed all of the patient examinations. The central corneal thickness (CCT) was measured using specular microscopy (SP3000P; Topcon Corporation, Itabashi-ku, Japan). The IOP was measured using Goldmann applanation tonometry.

### Statistical Analysis

The data were analyzed using JMP Pro 14 (SAS Institute, Cary, NC). The independent *t*-test and χ^2^ test for independent samples were used to analyze statistical differences between the groups. The cumulative probabilities of success were analyzed using the Kaplan–Meier survival curve and log-rank test. Pearson's correlation was used to analyze the relationship between two factors, and the coefficients of determination (*R^2^*) were calculated in a linear regression model. Difference tests for comparisons were considered significant at *P* < 0.05.

## Results

Twenty-five eyes with POAG and 15 eyes with XFG met the selection criteria and were evaluated in the current study. The demographics did not differ significantly between the two groups (Table 1). The rate of postoperative choroidal detachment in the XFG group was significantly higher (*P* = 0.004) than in the POAG group. Other complications such as hyphema, shallow anterior chamber, and iris or corneal contact with the Ex-PRESS were similar between the two groups (Table 1).

**Table 1.** Demographic Data for the Study Patients With POAG and XFG

Variable	POAG	XFG	*P*
Eyes/subjects, *n*	25/25	15/15	—
Right eye/left eye, *n*	10/15	8/7	0.41
Male/female, *n*	17/8	8/7	0.35
Age (yr), mean ± SD	69.4 ± 5.1	73.0 ± 5.1	0.08
Medically controlled IOP (mmHg), mean ± SD	24.6 ± 7.7	27.7 ± 10.8	0.29
Corneal endothelial cells per mm^2^, mean ± SD	2587 ± 405	2387 ± 518	0.18
CCT (µm), mean ± SD	509.9 ± 35.5	505.6 ± 36.6	0.71
Medication score, mean ± SD	4.4 ± 0.8	4.3 ± 0.9	0.81
Administration of anticoagulants, *n* eyes (%)	5 (20)	3 (20)	1
History of trabeculotomy, *n* eyes (%)	1 (4)	0 (0)	0.43
History of PEA + IOL, *n* eyes (%)	6 (24)	6 (40)	0.28
Combined with PEA, *n* eyes (%)	18 (72)	8 (53.3)	0.25
Complications, *n* eyes (%)			
Hyphema	6 (24)	1 (6.6)	0.16
Shallow anterior chamber	3 (12)	5 (33.3)	0.1
Choroidal detachment	2 (8)	7 (46.6)	0.004^**^
Bleb leak within 2 weeks	0 (0)	1 (6.6)	0.37
Iris contact	4 (16)	4 (26.6)	0.66
Cornea contact	0 (0)	0 (0)	
IOP after Ex-PRESS (mmHg)			
1 mo	11.2 ± 4.4	15.3 ± 5.8	0.015^*^
3 mo	10.8 ± 3.5	13.2 ± 3.4	0.036^*^
6 mo	11.0 ± 4.0	15.8 ± 4.7	0.001^**^
IOP reduction before and after Ex-PRESS (mmHg)			
1 mo	13.4 ± 9.9	12.4 ± 13.3	0.77
3 mo	13.8 ± 9.5	14.4 ± 11.1	0.85
6 mo	13.6 ± 9.7	11.9 ± 11.9	0.62
IOP reduction rate before and after Ex-PRESS (%)			
1 mo	49.4 ± 25.0	36.8 ± 34.7	0.19
3 mo	50.9 ± 23.7	47.0 ± 21.0	0.59
6 mo	50.0 ± 25.4	37.3 ± 26.0	0.13
Medication score after Ex-PRESS			
1 mo	0.04 ± 0.2	0.06 ± 0.2	0.71
3 mo	0.04 ± 0.2	0.33 ± 0.81	0.09
6 mo	0.2 ± 05	0.73 ± 1.0	0.03^*^

PEA, phacoemulsification and aspiration.

* *P* < 0.05.

** *P* < 0.01.

### Postoperative IOP, Medication Score, and Prognosis

**Figure 2.**
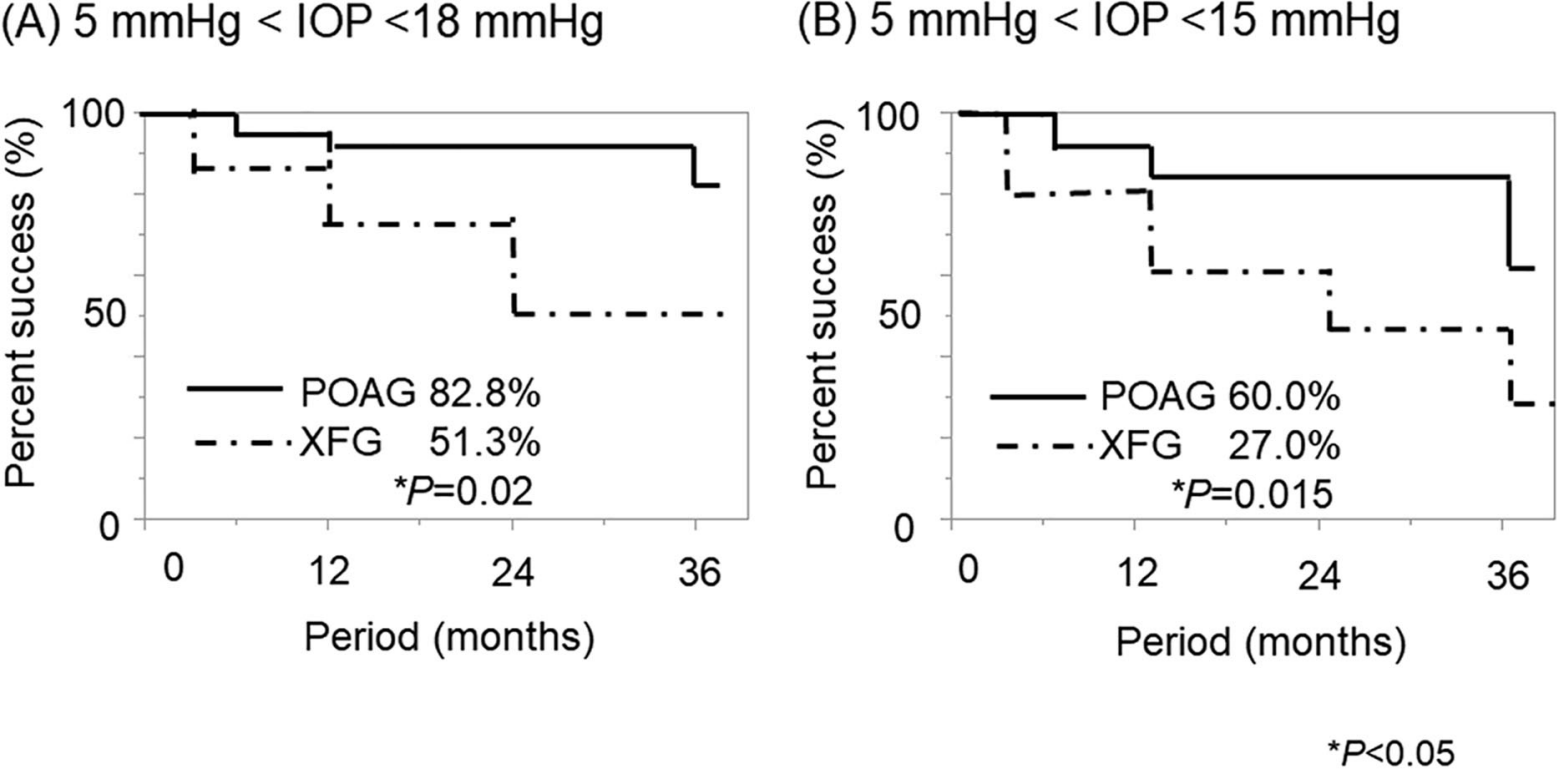
The cumulative probability of success defined as criterion A (5 mmHg < IOP < 18 mmHg and >20% IOP reduction with or without medication) and criterion B (5 mmHg < IOP < 15 mmHg and >20% IOP reduction with or without medication) after Ex-PRESS surgery in the POAG and XFG groups. The success rate was significantly higher in the POAG group than the XFG group for both criteria at 3 years postoperatively using the log-rank test.

The IOP was significantly higher in the XFG group at 1, 3, and 6 months postoperatively than in the POAG group (*P* = 0.015, *P* = 0.036, and *P* = 0.001, respectively). The medication score was significantly higher (*P* = 0.03) in the XFG group 6 months postoperatively than in the POAG group (Table 1). Furthermore, the surgical success rate at 3 years was significantly higher in the POAG group than in the XFG group for criterion A (*P* = 0.02; 5 mmHg < IOP < 18 mmHg, and >20% IOP reduction with or without medication) and criterion B (*P* = 0.015; 5 mmHg < IOP < 15 mmHg and >20% IOP reduction with or without medication) (Fig. 2).

### Differences in Intrableb Structural Changes Between the Groups

The fluid-filled space volume at 3 and 6 months (*P* = 0.005 and *P* = 0.022, respectively) and the total bleb volume at 6 months (*P* = 0.04) postoperatively decreased significantly in the XFG group compared with the POAG group (Table 2). In addition, the volume ratio of the low-scattering wall to the bleb wall (*P* = 0.028) at 6 months was smaller in the XFG group than in the POAG group (Table 3).

**Table 2.** Intrableb Volume and Group After Ex-PRESS Surgery

Variable	Postoperative Months	POAG	XFG	*P*
Total bleb	1	11.56 ± 1.71	11.99 ± 2.11	0.493
	3	12.31 ± 1.76	11.41 ± 1.76	0.126
	6	12.55 ± 2.75	10.85 ± 1.84	0.040^*^
Bleb wall	1	9.24 ± 2.02	10.11 ± 2.06	0.201
	3	9.56 ± 1.41	10.14 ± 1.71	0.247
	6	9.55 ± 1.38	9.45 ± 1.49	0.822
High-scattering wall	1	3.30 ± 1.05	3.74 ± 1.07	0.207
	3	3.58 ± 0.84	3.88 ± 0.65	0.251
	6	3.36 ± 1.17	3.88 ± 0.77	0.136
Medium-scattering wall	1	2.71 ± 0.54	2.50 ± 0.40	0.201
	3	2.62 ± 0.83	2.53 ± 0.55	0.688
	6	2.54 ± 0.72	2.57 ± 0.75	0.909
Low-scattering wall	1	3.22 ± 0.89	3.86 ± 1.29	0.072
	3	3.34 ± 0.82	3.73 ± 1.17	0.223
	6	3.64 ± 1.11	2.99 ± 0.97	0.068
Reticulated wall	1	5.94 ± 1.22	6.36 ± 1.51	0.337
	3	5.97 ± 1.14	6.26 ± 1.39	0.477
	6	6.18 ± 1.32	5.56 ± 1.52	0.181
Fluid-filled space	1	2.31 ± 1.40	1.87 ± 1.10	0.302
	3	2.75 ± 1.87	1.26 ± 0.66	0.005^**^
	6	2.99 ± 2.40	1.39 ± 1.29	0.022^*^

Data are expressed as mean ± SD. The reticulated wall is comprised of the medium- and low-scattering walls.

* *P* < 0.05.

** *P* < 0.01.

**Table 3.** Volume Ratio to Bleb Wall and Group After Ex-PRESS Surgery

Variable	Postoperative Months	POAG	XFG	*P*
High-scattering wall	1	0.35 ± 0.07	0.366 ± 0.10	0.557
	3	0.37 ± 0.07	0.38 ± 0.05	0.63
	6	0.35 ± 0.11	0.41 ± 0.08	0.07
Medium-scattering wall	1	0.30 ± 0.06	0.25 ± 0.07	0.054
	3	0.27 ± 0.07	0.25 ± 0.04	0.26
	6	0.26 ± 0.08	0.26 ± 0.04	0.962
Low-scattering wall	1	0.34 ± 0.05	0.37 ± 0.06	0.152
	3	0.34 ± 0.06	0.36 ± 0.06	0.532
	6	0.37 ± 0.09	0.31 ± 0.07	0.028^*^
Reticulated wall	1	0.64 ± 0.07	0.63 ± 0.10	0.557
	3	0.62 ± 0.07	0.61 ± 0.05	0.632
	6	0.64 ± 0.11	0.58 ± 0.08	0.07

Data are expressed as mean ± SD. The reticulated wall is comprised of the medium- and low-scattering walls.

* *P* < 0.05.

### Relationship Between Intrableb Measurements and IOP in Both Groups

**Figure 3.**
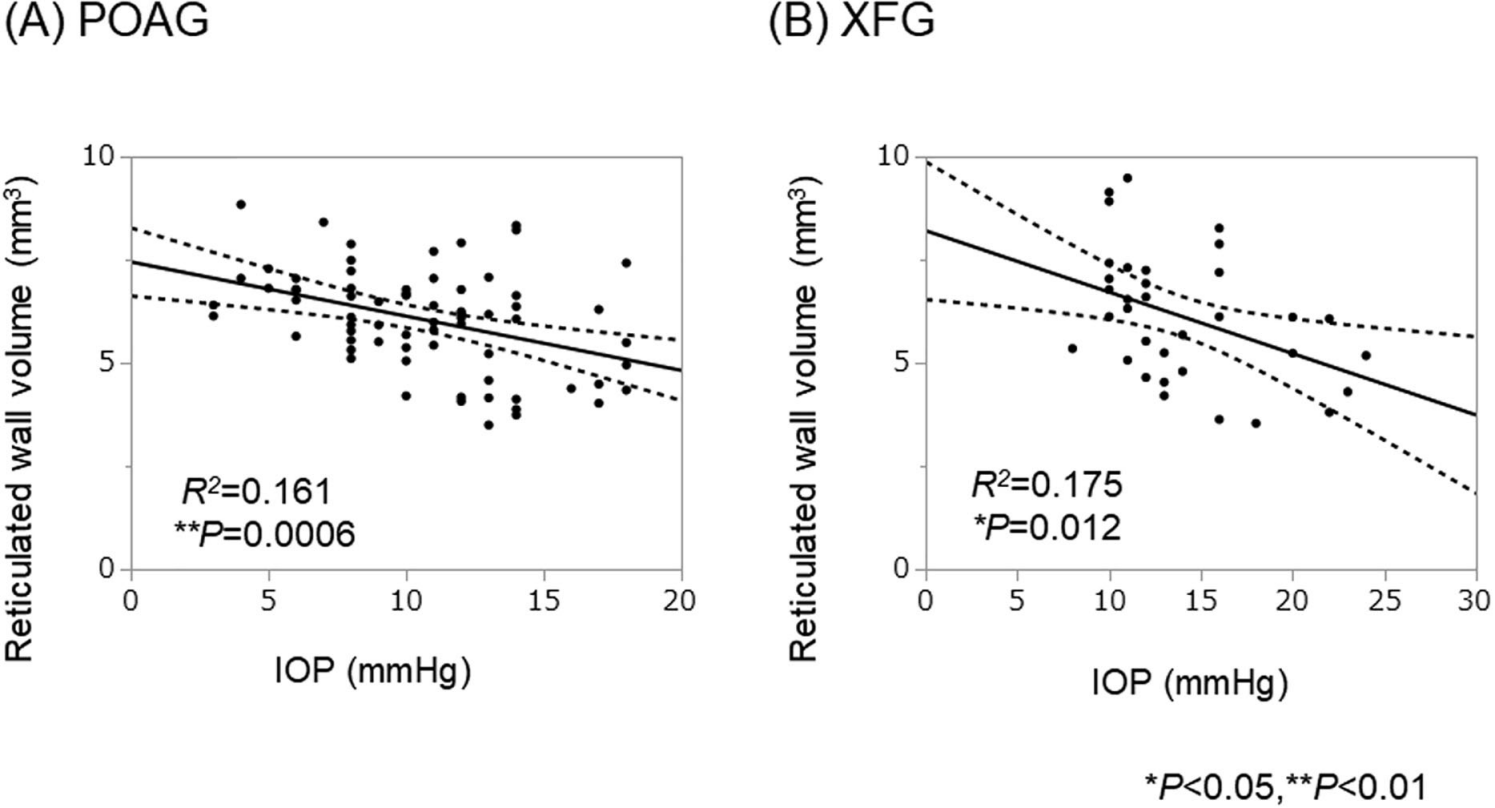
The relationship between the reticulated bleb wall volume and IOP in the POAG group and the XFG group. The reticulated wall volume was correlated negatively with the IOP in both groups without the use of other additional glaucoma treatment.

**Figure 4.**
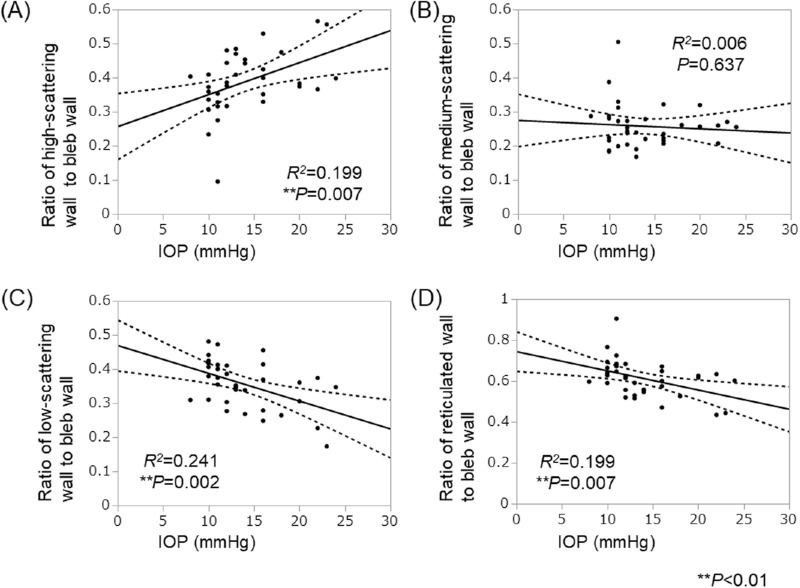
The relationship between the bleb wall volume ratio and IOP in XFG. The relationship between the bleb wall volume ratio of the bleb measurements and IOP is shown in the XFG group without the use of additional glaucoma treatment. (A) The volume ratio of the high-scattering wall to the bleb wall was correlated positively with the IOP. (B) The volume ratio of the medium-scattering wall to the bleb wall was not correlated with the IOP. (C) The volume ratio of the low-scattering wall to the bleb wall was correlated negatively with the IOP. (D) The volume ratio of the reticulated wall, which is comprised of the medium- and low-scattering walls to the bleb wall, was negatively correlated with the IOP.

The reticulated wall volume was correlated negatively with the IOP in both groups (Table 4, Fig. 3). The bleb wall volume also was correlated negatively with the IOP in the POAG group but not in the XFG group. In contrast, the volume ratio of the high-scattering wall to the bleb wall was correlated positively (*R^2^* = 0.199, *P* = 0.007) with the IOP in the XFG group but not in the POAG group. Furthermore, the volume ratio of the low-scattering wall and the reticulated wall to the bleb wall was correlated negatively (*R^2^* = 0.241, *P* = 0.002) with the IOP in the XFG group but not in the POAG group (Table 4, Fig. 4).

**Table 4.** Relationship Between Bleb Volume Measurements and IOP in Two Groups After Ex-PRESS Surgery

	IOP in POAG			IOP in XFG		
Variable	Slope	*R* *^2^*	*P*	Slope	*R* *^2^*	*P*
Intrableb volume						
High-scattering wall	−0.041	0.023	0.211	0.045	0.052	0.184
Medium-scattering wall	−0.064	0.121	0.003^**^	−0.31	0.051	0.189
Low-scattering wall	−0.066	0.066	0.032^*^	−0.117	0.167	0.014^*^
Fluid-filled space	0.110	0.046	0.076	−0.015	0.003	0.745
Total bleb	−0.062	0.011	0.373	−0.118	0.063	0.144
Bleb wall	−0.173	0.161	0.0006^**^	−0.103	0.061	0.15
Reticulated wall	−0.131	0.161	0.0006^**^	−0.148	0.175	0.012^*^
Volume ratio to bleb wall						
High-scattering wall	0.002	0.01	0.402	0.009	0.199	0.007^**^
Medium-scattering wall	−0.001	0.004	0.569	−0.001	0.006	0.637
Low-scattering wall	−0.001	0.003	0.638	−0.008	0.241	0.002^**^
Reticulated wall	−0.002	0.01	0.402	−0.009	0.199	0.007^**^

The bleb wall is comprised of the high-, medium-, and low-scattering walls. The reticulated wall is comprised of the medium- and low-scattering walls.

* *P* < 0.05.

** *P* < 0.01.

## Discussion

In XFG, a vision-threatening form of secondary glaucoma worldwide, controlling the IOP is more difficult than in POAG after filtration surgery.^4^^–^^6^ The current study evaluated the early intrableb structural changes in POAG eyes and XFG eyes after Ex-PRESS surgery in detail using 3D AS-OCT and demonstrated the differences in the intrableb wound-healing process between them.

### Decreasing Fluid-Filled Volume and Changes in the Bleb Wall in XFG

In the current study, the bleb volume decreased in the XFG group compared with the POAG group 6 months after Ex-PRESS surgery. Our results agreed with a previous report by Tojo et al.^18^ at 1 year after Ex-PRESS surgery. A more detailed evaluation showed that the fluid-filled space volume was smaller in the XFG group compared with the POAG group 3 and 6 months postoperatively (Table 2). Furthermore, the volume ratio of the low-scattering wall to the bleb wall also was significantly smaller in the XFG group than in the POAG group at the same time points (Table 3). Previous studies have reported that thick low-scattering walls contain aqueous humor, suggesting a functional filtration bleb.^19^^–^^21^^,^^23^^,^^24^ Therefore, our results suggest that the filtration volume of XFG decreases in the early postoperative period compared with POAG. In the current study, the rate of choroidal detachment was high in the XFG group early postoperatively, suggesting that underfiltration due to decreased production of aqueous humor may have caused the bleb failure and increased the IOP. Our results were supported by the previous report of Narita et al.,^23^ who reported that the volume ratio of the low-scattering wall to the bleb wall was smaller in the unsuccessful group in which a low IOP could not be maintained compared with the successful group 1 year after trabeculectomy.

### Relationship Between Bleb Measurements and IOP

We first considered the relationship between the entire bleb and IOP because some studies have reported that the IOP is lower with a larger bleb. Tojo et al.^18^ reported a negative correlation between the bleb volume and IOP. Kawana et al.^21^ also reported that the bleb height was correlated negatively with the IOP. In contrast, Tominaga et al.^24^ reported that the bleb height was not correlated with the IOP. Our results are similar to the report of Tominaga et al.^24^ because the total bleb volume was not correlated with the IOP in the POAG and XFG groups (Table 4). This can be explained by the high IOP in a case with an encapsulated bleb with a large fluid-filled space, even if the total bleb volume was large. In fact, the fluid-filled space volume was not correlated with the IOP in both groups in our study (Table 4).

We then considered the relationship between the bleb wall and IOP. Tominaga et al.^24^ reported that the bleb wall thickness was correlated negatively with the IOP. Our study showed a negative correlation between the bleb wall volume and IOP in the POAG group, but not the XFG group, suggesting that the relationship between the structure of the bleb wall and IOP differed between the two groups (Table 4). In detail, the reticulated wall, which indicated the loose stromal connective tissue containing filtrated aqueous humor, was correlated negatively with the IOP throughout 6 months after Ex-PRESS surgery in both groups (Table 4, Fig. 3). These results indicate that patients with low IOP tended to have larger reticular volumes in common with both groups, and they agree with a previous report of Kawana et al.^21^ that the reticulated wall volume was significantly negatively correlated with the IOP after trabeculectomy in several glaucoma types. In contrast, we found that the volume ratio of the reticulated wall to the bleb wall was correlated negatively with the IOP, and the volume ratio of the high-scattering wall to the bleb wall was correlated positively with the IOP in the XFG group but not the POAG group (Table 4, Fig. 4). Our retrospective study partially agrees with a prospective study by Watanabe-Kitamura et al.^25^ that reported that the ratio of the high-scattering wall to the bleb wall was correlated positively with the IOP in 11 POAG eyes and 11 XFG eyes 12 months after trabeculectomy. The reticulated wall is considered to be the loose connective tissue that contains the aqueous humor, whereas the high-scattering wall is the dense stromal connective tissue that is the fibrosis formed by scarring.^19^^–^^21^^,^^23^^,^^24^ Thus, the decreased filtration and increased IOP due to fibrosis of the bleb wall were more prominent in XFG than in POAG in the current study. From these results, the relationship between the volume ratios of the high- or low-scattering wall to the bleb wall and IOP differed in the POAG and XFG groups.

Several possible factors may account for the differences between our results and other reports, such as glaucoma type, surgical procedure, measurement time point, measurement method, and effect of additional eye drop treatment postoperatively. For example, we focused on POAG and XFG, performed Ex-PRESS surgery with a fornix-based conjunctival flap, and analyzed the morphology in a limited region of the bleb centered on the scleral flap up to 6 months postoperatively. In the study of Tojo et al.,^18^ the surgical procedure and glaucoma types were similar to those of the current study, but those authors evaluated patients 1 year or more postoperatively, and several patients had markedly high IOP. Narita et al.^23^ performed trabeculectomy with a fornix-based conjunctival flap for several glaucoma types and evaluated patients 1 year postoperatively. Kawana et al.^21^ performed trabeculectomy with a limbal-based conjunctival flap for several glaucoma types, and they evaluated patients at different time points postoperatively. For the measurements, we automatically categorized 3D bleb images into four scattering intensities, as did Narita et al.,^23^ whereas Tominaga et al.^24^ evaluated two-dimensional bleb images manually. In addition, Kawana et al.^21^ analyzed the reticular volume and fluid-filled space in manually classified 3D bleb images. Tojo et al.^18^ evaluated the hyporefractive area as the bleb volume using 3D bleb images without analyzing the intrableb structure in detail.

Furthermore, additional postoperative treatment with eye drops is also considered to affect bleb formation. We excluded cases that needed additional medication to evaluate the relationship between bleb measurements and IOP. The *R*^2^ values were not large, suggesting that other factors are associated with the bleb wall besides IOP (Figs. 3, 4).

### Difficulty Maintaining Bleb Formation in XFG

In the current study, the XFG group had higher IOP due to lower filtration of aqueous humor and progressive fibrosis of the bleb wall in the early postoperative period compared with the POAG group, which may reflect the lower surgical success rate at the 3-year evaluation. The difference between the two groups in the production of various bioactive substances that promote fibrosis in the bleb may have affected this result. For example, high aqueous autotaxin and lysophosphatidic acid levels induced fibrotic changes in the bleb in XFG eyes after filtration surgery.^27^^,^^28^ It also has been reported that monocyte chemoattractant protein-1 (MCP-1), a proinflammatory cytokine, increases significantly in XFG eyes compared with POAG eyes with a history of cataract surgery^29^ and that high MCP-1 levels result in bleb failure.^30^ In fact, numerous eyes had IOL implantation postoperatively in our study, suggesting high aqueous MCP-1 levels in the XFG group.

### Ex-PRESS Surgery for XFG

Ex-PRESS surgery may have some advantages for XFG eyes. A fibrotic response and hyphema after trabeculectomy have been reported to occur more frequently in XFG.^31^^–^^33^ In contrast, Ex-PRESS surgery is less invasive with fewer complications than trabeculectomy because it does not require iridectomy and sclerotomy.^34^^,^^35^ In fact, only one case of hyphema developed in XFG eyes in this study. In addition, the outflow rate is constant, and it is generally considered that a shallow anterior chamber and choroidal effusion are unlikely to develop. However, in the current study, the percentage of choroidal detachment was high, which may have affected the surgical outcome. A disadvantage is that the numbers of corneal endothelial cells decrease in XFG eyes.^35^ In addition, needling under the scleral flap may be difficult because the device is inserted. Therefore, there are advantages and disadvantages for both trabeculectomy and Ex-PRESS surgery. Further study is needed to determine suitable cases for each surgical procedure in XFG eyes.

The current study had several limitations. First, this study was hospital based and retrospective. Second, this study is specific to the Ex-PRESS device that three surgeons implanted. We think that the minimally invasive Ex-PRESS surgery can eliminate bias in contrast to trabeculectomy because similar surgical procedures were performed among the surgeons. Third, more cases should be added in the future to study the results in detail because the number of current cases was limited. However, the association may be sufficiently strong to be considered significant despite the small sample size. Fourth, the blebs were evaluated in the same limited area because of the difficulty measuring the entire bleb.

In summary, we evaluated the intrableb scattering intensity of OAG in the early postoperative period after Ex-PRESS surgery. The results suggest that eyes with XFG have a different course of structural changes in the bleb wall and fluid space compared with eyes with POAG, probably due to unstable filtration and fibrosis in the bleb. Difficulty in early bleb formation results in poor short-term IOP control in XFG.
